# Le syndrome de Miller Fischer: à propos d’un cas pédiatrique

**DOI:** 10.11604/pamj.2018.30.37.14136

**Published:** 2018-05-17

**Authors:** Noureddine Khiri, Moussa Lazreg, Houssam Bkiyar, Soufiane Diyas, Brahim Housni

**Affiliations:** 1 Service de Réanimation, CHU Mohamed VI, Faculté de Médecine et de Pharmacie d’Oujda, Université Mohammed 1^er^ Oujda, Maroc

**Keywords:** Syndrome de Miller Fischer, forme pédiatrique, détresse respiratoire, immunoglobuline intraveineuses, Miller Fischer syndrome, paediatric form, respiratory distress, intravenous immunoglobulin

## Abstract

Le syndrome de Miller Fisher (MF) représente une forme rare du syndrome de Guillain-Barré qui ne survient que dans 5% des cas chez l'adulte alors que les cas pédiatriques décrits dans la littérature sont encore plus rares. Nous présentons le cas d'un enfant de 12 ans chez qui on a posé le diagnostic de syndrome de Miller Fischer en se basant sur la présentation clinique avec une atteinte oculaire en premier plan et la positivité des anticorps anti-ganglioside. Notre patient est admis dans notre structure dans un tableau de détresse respiratoire nécessitant l'intubation orotrachéale et le recours aux immunoglobulines intraveineuses avec bonne évolution clinique.

## Abstract (en)

Miller Fischer (MF) syndrome is a rare variant of Guillain-Barré syndrome which occurs only in 5% of cases among adults while the pediatric cases described in the literature are even more rare. We here report the case of a 12-year old boy who was diagnosed with Miller Fischer syndrome based on clinical presentation characterized by obvious ocular involvement and anti-ganglioside antibody-positivity. The patient was admitted to our hospital with respiratory distress requiring orotracheal intubation and intravenous immunoglobulins, with good clinical evolution.

## Introduction

Le syndrome de Miller Fisher (MF) est une variante du syndrome de Guillain-Barré qui n'en présente qu'une minorité rare de 5%. Ce syndrome est d'autant plus rare chez l'enfant [1]. Les formes pédiatriques sont souvent modérées en termes de gravité avec une atteinte oculaire en premier plan et ne cause que rarement l'insuffisance respiratoire ou le coma transitoire [2]. Le pronostic est souvent favorable et la guérison est très souvent spontanée [3]. Les traitements immunomodulateurs n'ont montré que peu d'influence sur le pronostic, cependant un traitement par immunoglobuline paraît améliorer le pronostic des malades ayant nécessité une intubation [2]. Nous rapportons un cas de syndrome de Miller Fischer chez un enfant de 12 ans.

## Patient et observation

Il s'agit d'un patient âgé de 12 ans, issu d'un mariage consanguin de premier degré, ne présentant aucun antécédent particulier. Il est admis dans notre structure pour détresse respiratoire faisant suite à une symptomatologie neurologique d'installation brutale faite de diplopie horizontale, de blépharoptose bilatérale, d'amimie de la face, de dysphonie, de difficulté à la déglutition avec paresthésies des membres supérieurs. L'examen clinique a trouvé un enfant en insuffisance respiratoire aigüe avec cyanose et toux inefficace ayant nécessité une intubation orotrachéale et mise sous ventilation mécanique en urgence après sédation. L'examen neurologique a révélé une marche et position debout difficile, une parésie des deux membres supérieurs avec des réflexes ostéo-tendineux déprimés, un déficit prédominant au niveau des ceintures, et une atteinte bilatérale des nerfs oculomoteur commun III, oculomoteur externe VI, du nerf facial VII, et des nerfs mixtes (IX, X, XI). L'imagerie par résonance magnétique cérébrale est revenue normale. L'analyse du liquide cérébro-rachidien n'a pas trouvé de dissociation cyto-protéinorachique. Les anticorps anti-gangliosides (anti-GM1, anti-GD 1b, anti-GQ 1b) ont été positifs. L'électromyogramme a révélé une démyélinisation. Le patient a reçu une première cure d'immunoglobuline de 15 g/j pendant cinq jours, débutée le lendemain de son admission. Au bout de 5 jours, le patient est trachéotomisé et respire spontanément avec saturation normale à l'air ambiant et présente une bonne évolution clinique avec régression du déficit moteur des membres supérieurs, ainsi qu'au niveau de la face avec disparition du ptosis.

## Discussion

Le syndrome de MF est rapporté dans seulement 5 à 10% des cas du syndrome de Guillain-Barré de l'adulte. Chez l'enfant, les cas décrits sont encore plus rares [1]. L'atteinte oculaire; notamment la diplopie et l'ataxie se présentent le plus souvent comme les premiers symptômes dans 38,6% et 20,6% des cas respectivement [4]. Une étude de Berlit et al a objectivé une atteinte de nerfs autre que le nerf III comme chez notre patient. L'atteinte du nerf VII a lieu dans 45,7%, le nerf IX et le X dans 39,9% des cas et le nerf XII dans 13% des cas [4]. Alors que l'atteinte périphérique est la règle, l'atteinte centrale est exceptionnelle [5]. Le syndrome de MF peut être fatal en entrainant une insuffisance respiratoire ou un coma transitoire, mais ces deux formes sont exceptionnelles [2]. Les études immunologiques sur le syndrome MF par les anticorps monoclonaux anti-GQ1b ont montré un rôle pathogénique direct des anticorps anti-GQb1 permettant ainsi d'expliquer les symptômes rencontrés. La positivité de ces anticorps permet un diagnostic positif vu sa sensibilité qui arrive jusqu'à 90% [3,6]. De nombreuse séries de patients atteints de ce syndrome traités par immunothérapie faisant recours à la plasmaphérèse et/ou à l'immunoglobuline intraveineuse ont échoué à montrer le moindre bénéfice de l'immunothérapie [7-9], cependant un traitement par immunoglobuline paraît améliorer le pronostic des malades ayant nécessité une intubation, ce qui a été aussi retrouvé chez notre patient [2]. Le pronostic est souvent favorable et la guérison est très souvent spontanée [3]. Dans l'étude menée par Berlit et al, la guérison survient après une moyenne de 10,1 semaines. Des symptômes résiduels peuvent persister dans 33,2% des cas avec possibilité de récidives et le décès est possible quoique rarement par insuffisance respiratoire ou coma suite à une atteinte nerveuse centrale [4].

## Conclusion

Le syndrome MF est une forme rare qui ne présente que 5% du syndrome de Guillain-Barré. Les formes pédiatriques sont beaucoup plus rare que chez l'adulte, souvent moins sévère car ne se complique que rarement d'insuffisance respiratoire. Le traitement par plasmaphérèse et/ou immunoglobuline intraveineuse n'a généralement de rôle que chez des malades se présentant pour une détresse respiratoire et ayant nécessité une intubation. Le pronostic est souvent favorable avec très peu de séquelles et un taux de mortalité très bas.

## Conflits d’intérêts

Les auteurs ne déclarent aucun conflit d'intérêts.
